# Genome-Wide Analysis of BpYABs and Function Identification Involving in the Leaf and Silique Development in Transgenic Arabidopsis

**DOI:** 10.3390/ijms23031670

**Published:** 2022-01-31

**Authors:** Feng Tang, Dan Zhang, Naizhi Chen, Xianjun Peng, Shihua Shen

**Affiliations:** 1 Key Laboratory of Plant Resources, Institute of Botany, The Chinese Academy of Sciences, Beijing 100093, China; tangfeng76@163.com (F.T.); danzhang@Mabplex.com (D.Z.); chennaizhi@ibcas.ac.cn (N.C.); 2 College of Life Sciences, University of Chinese Academy of Sciences, Beijing 100049, China

**Keywords:** paper mulberry, adaxial–abaxial polarity, lateral organ development, pods and seeds development

## Abstract

YABs play an important role in the leaf development of the paper mulberry (*Broussonetia papyrifera*) and of the heterophylly. Thus, we investigated the function of BpYABs. Gene cloning, phylogenetic analysis, motif identification, subcellular localization, transactivation activity assay, qRT-PCR, in situ hybridization, and ectopic expression were used in our study. Six *BpYAB*s were isolated, and four of them had transcriptional activity. BpYAB1, BpYAB3, BpYAB4, and BpYAB5 were localized to the nucleus. *BpYAB1* was only expressed in the flower, while *BpYAB6* was not expressed in any detected tissues; the four remaining *BpYAB*s were expressed in the bud, leaf and flower, and their expression level decreased with leaf development. Further in situ hybridization showed that *BpYAB3* and *BpYAB5* were expressed in the vascular tissues and lamina, but neither showed the adaxial–abaxial polarity distribution pattern in the mature leaf lamina. Ectopic expression of *BpYAB2*, *BpYAB3*, *BpYAB4* and *BpYAB5* induced increased expression of *AtWOX1* and caused the leaf of Arabidopsis to become smaller and curl downwards. Ectopic expression also led to shorter siliques and smaller seeds, but not for *BpYAB5*. These results suggest that BpYABs have functional divergency and redundancy in regulating leaf and silique development.

## 1. Introduction

YABBY is a plant-specific transcription factor family containing a zinc-finger domain and a YAB domain [1]. The YAB domain functions in DNA binding, and the zinc-finger domain regulates the dimerization between the YABBYs and other proteins [2]. *YAB* genes play critical roles, mainly in lateral organ development, such as leaf and flower morphogenesis, carpel polarity establishment, lamina outgrowth, and leaf polarity maintenance [3]. Most of the YABBYs in different species are localized in the nucleus. Four YABBYs (OsYAB1, OsYAB3, OsYAB4 and ObSH3) in rice [4,5,6,7], two YABBYs (VpYAB1 and VpYAB2) in grape [8], IaYAB2 [9], BraYAB1 [10], and MsYAB5 [11], are obvious nuclear localization proteins.

In gymnosperms, the YABBYs are grouped into four clades, called A, B, C, and D, while they are divided into five subfamilies in angiosperms, named *FIL*-like, *YAB2*-like, *YAB5*-like, *CRC*-like, and *INO*-like [12]. Reports from core eudicots suggested the evolution of the YABBY family coincides with the origin of leaves in seed plants, and YABBY activity is required for initiating the lamina and maintenance of leaf polarity, not the initial establishment of polarity [13]. Hence, it is necessary to explore the evolutionary and functional differences of *YABBY* genes in plant species.

There are six *YABBY* genes in *Arabidopsis thaliana*: *FIL*, *YABBY2*, *YABBY3*, *YABBY5*, *CRC* and *INO*. The *CRC* gene is essential for carpel polarity establishment and nectary specification in *A. thaliana* [14], while *INO* expresses in the outermost cell layer of the ovule and promotes outer integument growth [15]. The other four *AtYAB* genes have redundant functions in repressing the shoot apical meristem (SAM) and activating laminar development [16,17]; thus, *YABBY* is considered to be the candidate gene for the evolution of stem-to-leaf transformation [18]. Ectopic SAM and axillary meristems are formed in the *fil yab3* mutant, and the stem cell activity-associated gene *CLAVATA3* (*CLV3*) and *WUSCHEL* (*WUS*) are expanded in the *fil yab3* double mutant [19]. Therefore, *YABBY* genes have a great impact on the meristem maintenance, indicating the non-cell-autonomous activity.

The non-cell-autonomous activity of *YABBY* shows defects in the adaxial domain of the leaf, where the *YABBY* gene is not expressed [20]. There are eight YABBY members in the monocot model plant rice (*Oryza sativa* L.), and they are mainly involved in reproductive organ development [3]. *OsYAB1* is highly expressed in the stamen and carpel primordia but is not distributed in a polar manner [4,5]. Loss of function of *TOB1* results in various defects in spikelet development, and *TOB1* does not show a polar expression pattern localized to the abaxial domain in rice [21]. Compared with the abaxially localized expression of *CRC* in developing carpels, the *DROOPING LEAF* (*DL*) gene is expressed in the carpel primordia and plays a crucial role in carpel specification and modulating leaf midrib development [22]. In maize, the homologous gene of *Arabidopsis FIL* and rice *TOB1* is expressed in the adaxial region of the leaf primordia [23]. Recent research has shown that the *YABBY* gene may play a role in seed shattering in cereals. Loss-of-function of the *Shattering1* (*Sh1*) gene, the homologous gene of *OsYAB2* (*OsSh1*), gives rise to the non-shattering seed phenotype in domesticated sorghum [24]. *ObSH3*, a YABBY transcription factor in *O. barthii*, is required for the development of the seed abscission layer [7]. Altogether, the function of *YABBY* genes is diversified in different species. In Arabidopsis, *FIL*, *YAB2*, *YAB3*, and *YAB5* transcripts are detected in the abaxial side of primordia, determining the abaxial cell fate, the *CRC* gene is expressed abaxially in the carpel, and *INO* mRNA is detected in the abaxial epidermis of the outer integument. However, the *OsYAB* genes are not expressed in a polar pattern and do not promote abaxial or adaxial polarity in lateral organs. The expression pattern of *YABBYs* between monocots and dicots indicates that *YABBY* genes are involved in the process of angiosperm diversification, and the functions of *YABBY* genes are seemingly diversified in seed plants, but how they function in woody plants remains unclear.

The paper mulberry (*Broussonetia papyrifera*) belongs to the Moraceae family and is a perennial woody tree. As a model plant, the paper mulberry can be used to explore the mechanism of lignin synthesis, nitrogenous and flavonoid metabolism, plant resistance, heterophylly formation, and other key issues [25], and more importantly, the resolution of the paper mulberry genome will facilitate these explorations [26]. As *YABBYs* play a critical role in the morphogenesis establishment of lateral organs in many species, the study of *YABBYs* in paper mulberry is interesting and important. The expression level of three *YABBY* genes in paper mulberry are inhibited after 6 h cold treatment, suggesting that they may function in growth regulation under cold stress [27]. Here, we identified *YABBY* genes from the paper mulberry genome and analyzed their dynamic expression profiles in different developmental stages of the leaf. Furthermore, we identified the function of four *BpYAB*s in Arabidopsis. The results provide essential information about the *BpYAB* family and contribute to understanding the role of *YABs*.

## 2. Results

### 2.1. Identification and Phylogenetic Analysis of the YABBY Gene Family in Paper Mulberry

A total of six YABBY members were identified from the genome of paper mulberry and named BpYAB1 to BpYAB6, according to the YABBYs in Arabidopsis. *BpYAB2*, *BpYAB3*, *BpYAB4* and *BpYAB6* were located in chromosome 9, chromosome 6, chromosome 1 and chromosome 7, respectively. *BpYAB1* was located in scaffold094 and *BpYAB5* in scaffold146 (Figure S1). The directions of *BpYAB2* and *BpYAB3* were reverse, while *BpYAB4* and *BpYAB6* were forward in the genome. The molecular weight of BpYAB proteins was between 20 and 25 kD (Table 1), and the predicted isoelectric point of each BpYAB protein ranged from 4.97 to 9.32. The relative genomic locations of the six YABBY members and other information are shown in Figure S1 and Table 1.

**Figure 1:**
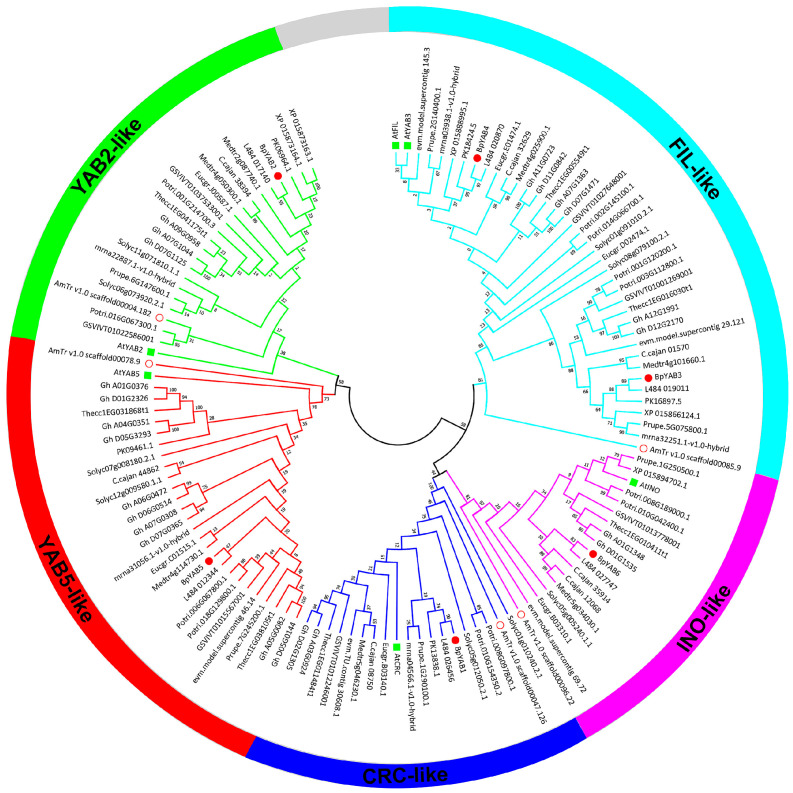
BpYABs clustered into five groups by phylogenetic analysis. The phylogenetic tree of YABBYs is divided into five clades, named *FIL*-like, *CRC*-like, *INO*-like, *YAB2*-like and *YAB5*-like, containing 127 amino acid sequences from 17 species. The paper mulberry (red filled circle) has two members in the *FIL*-like group, and other four groups contain only one member. The YABBYs with the green boxes are from *A. thaliana* and the red circles are from *A. trichopoda*. All the YABBY proteins were aligned by MUSCLE in MEGA 7.0 and adjusted manually.

In order to explore the evolutionary relationships among *YABBY* genes, a neighbor-joining tree based on 17 species, including 127 protein sequences, was constructed (Figure 1). All species were from dicotyledons, including the base species of angiosperms, *Amborella trichopoda*, and the protein sequences are displayed in Table S1. The results indicated that all members of YABBY were divided into five clades, named *FIL*-like, *INO*-like, *CRC*-like, *YAB2*-like and *YAB5*-like. There were five *YABBY* genes in *A. trichopoda* belonging to the five subfamilies (Figure 1), indicating that *YABBY* members from other species in the five subfamilies might have originated from these five *YABBY* genes in *A. trichopoda*. Six *YABBY* members of paper mulberry were also categorized into five subfamilies. Both *BpYAB3* and *BpYAB4* belonged to the *FIL*-like clade, while *BpYAB2* and *BpYAB5* belonged to the *YAB2*-like and *YAB5*-like clades, respectively. These four *BpYAB* genes may participate in leaf development according to the function of their homologous gene in Arabidopsis. *BpYAB1* belonged to the *CRC*-like clade, implying that *BpYAB1* might be involved in flower development. *BpYAB6* belonged to the *INO*-like clade, suggesting that the function of *BpYAB6* might be associated with ovule development (Figure 1). The number of *YABBY* genes in the selected woody plants, *Prunus persica*, *Ziziphus jujuba*, paper mulberry, *M. notabilis* and *Eucalyptus grandis*, was five or six, indicating that *YABBY* members in woody plants did not undergo expansion during the species diversification and evolution. Among the five clades, the number of *FIL*-like groups was highest, at 39, and there were at least two members in each species except *A. trichopoda*. The numbers of the remaining four groups were 28, 23, 19, and 18, belonging to the *YAB5*-like, *YAB2*-like, *CRC*-like, and *INO*-like groups, respectively. It was interesting to find that both the *INO*-like and *CRC*-like clades contained only one gene in each species, implying that these two groups did not undergo expansion. The *FIL*-like, *YAB2*-like, and *YAB5*-like groups had obviously undergone expansion, but the expansion rate was relatively slow. We found that the *YABBY* genes in mulberry (*M. notabilis*) displayed a close relationship with paper mulberry (Figure 1). The number of *YABBY* genes in *A. thaliana*, paper mulberry, and *M. notabilis* was the same.

### 2.2. Sequence Analysis and Structural Characterization of BpYAB Members

The *BpYAB* gene sequences are displayed in Table S2. The length of *BpYAB* genes varied from 1 to 7 kb. The longest one was *BpYAB2* with 6482 bp, while the shortest was *BpYAB6*, with 1197 bp. Although their genomic length was over a large span, the coding sequences were all around 600 bp, and the protein length was about 200 amino acids (Table 1). *BpYAB2* and *BpYAB*6 had six exons, and the remaining *BpYABs* all had seven exons (Figure S2a). The lengths of exon 4 and exon 5, which were 49 and 76 bp, were the same in all *BpYABs* (Table S3). Each of the BpYAB proteins contained two highly conserved domains, the C_2_C_2_ and YABBY domains, and their positions were relatively consistent among the different BpYAB members (Figure S2b). In the nucleotide sequence coding, the C_2_C_2_ domain was distributed in part of exon 1 and exon 2, and the YABBY domain spread in exon 4, exon 5, and exon 6 (Figure S2a). The C_2_C_2_ domain of BpYAB proteins contained 43 amino acids, including 16 highly conserved amino acids. The length of the YABBY domain was 56 amino acids, including 28 highly conserved amino acids. The conserved amino acids are marked with asterisks in Figure S2c,d.

### 2.3. Analysis of Cis-Acting Elements in the Promoters of BpYAB Genes

The name and number of all the *cis*-elements detected in the *BpYAB* gene promoter regions are displayed in Table S4. We found that both the number and kinds of *cis*-elements in the promoter of *BpYAB1* were the most among six *BpYABs*. There were 529 *cis*-elements in 107 categories in the promoter of *BpYAB1*, of which the most numerous ones were CACTFTPPCA1 (42), DOFCOREZM (27), CAATBOX1 (23), ROOTMOTIFTAPOX1 (22), CURECORECR (22), CTRMCAMV35S (22), GT1CONSENSUS (18), GATABOX (18) and more. There were 429 *cis*-elements in 85 categories in the promoter of *BpYAB2*, of which the most numerous ones were CAATBOX1 (25), ARR1AT (24), CACTFTPPCA1 (22), CTRMCAMV35S (20), DOFCOREZM (20), ROOTMOTIFTAPOX1 (19), GT1CONSENSUS (17), etc. There were 509 *cis*-elements in 92 categories in the promoter of *BpYAB3*, of which the most numerous ones were DOFCOREZM (39), ROOTMOTIFTAPOX1 (33), CACTFTPPCA1 (30), CAATBOX1 (28), ARR1AT (20), GATABOX (18), MARTBOX (18), GT1CONSENSUS (17), etc. There were 469 *cis*-elements in 102 categories in the promoter of *BpYAB4*, of which the most numerous ones were GT1CONSENSUS (28), CACTFTPPCA1 (26), ARR1AT (25), CAATBOX1 (25), DOFCOREZM (24), ROOTMOTIFTAPOX1 (17), GTGANTG10 (16), GATABOX (15), etc. In the promoter of *BpYAB5*, there were 470 *cis*-elements in 94 categories, among which there were DOFCOREZM (48), POLLEN1LELAT52 (28), GT1CONSENSUS (23), ARR1AT (21), CAATBOX1 (19), CACTFTPPCA1 (19), ROOTMOTIFTAPOX1 (16), GATABOX (15), and so on. There were 491 *cis*-elements in 97 categories in the promoter of *BpYAB6*, among which the most numerous ones were CACTFTPPCA1 (41), DOFCOREZM (35), GT1CONSENSUS (25), CAATBOX1 (23), EBOXBNNAPA (22), MYCCONSENSUSAT (22), GATABOX (21), ARR1AT (18), GTGANTG10 (17), and so on. It can be seen from the above statistics that the number of *cis*-elements such as CACTFTPPCA1, DOFCOREZM, ROOTMOTIFTAPOX1, GT1CONSENSUS, ARR1AT and CTRMCAMV35S is the majority, which shows that BpYABs participates in these same regulatory processes, but the quantitative composition was different, indicating their ability to function was different.

Besides, we focused on three groups of these *cis*-elements related to development, hormone signaling, and environment response (Table 2). The RY-element, involved in seed-specific regulation, was found only in *BpYAB3*, and MSA-like, which functions in cell cycle regulation, was specific to *BpYAB6*. All the *BpYABs* had at least one hormone-related *cis*-element, indicating that the expression of *BpYAB* may be regulated by these hormones. The auxin-related *cis*-regulators, AuxRR-core and TGA-element, were predicted in *BpYAB4* and *BpYAB2* specifically, which implied that auxins may regulate these two genes. ABRE, the ABA-responsive element, was found in *BpYAB1*, *BpYAB3,* and *BpYAB4*, and seven of ABRE were in *BpYAB1* promoter. P-box and TATC-box are gibberellin (GA)-responsive elements and were found in four *BpYABs*. The salicylic acid (SA)-responsive element, the TCA-element, was found in *BpYAB2* and *BpYAB5*. Some promoters also contained several abiotic stress response elements. For instance, all *BpYABs* except for *BpYAB1* had the ARE *cis*-element, which is essential for anaerobic induction. LTR, a low-temperature-responsive *cis*-regulator, was only found in *BpYAB2*. As for the WUN-motif, the wound-responsive element was located in *BpYAB5* specifically (Table 2). In any case, the *cis*-element analysis illustrated that *BpYAB* genes participated in plant development, hormone signaling, and response to environmental stresses.

### 2.4. The Conserved Motif Identification of BpYAB Family

The phylogenetic relationships were reconstructed, and the conserved motifs of YAB proteins were compared among the three species (Figure S3). Consistently, these YABBYs were divided into five clades (Figure S3a). A total of ten motifs were observed (Figure S3b), and most of these motifs are not yet well-characterized. The sequences of the ten motifs are displayed in Figure S4. Motif 1 was located in the YABBY domain, while motif 2 was located in the C_2_C_2_ domain. Some motifs were only found in genes within the same clade. Motif 3 was found in three clades, *FIL*-like, *YAB2*-like, and *YAB5*-like, inferring that these three clades were closer in the evolutionary relationship. Motif 4 was only identified in the *FIL*-like clade in three species, and motif 9 was also specifically identified in the *FIL*-like clade, except in *AtYAB3*. Motifs 5, 6, 8, and 10 were found only in the paper mulberry and *M. notabilis*, revealing that these two species have a very close evolutionary relationship.

### 2.5. Transactivation Activity and Subcellular Localization of the BpYAB Family

**Figure 2:**
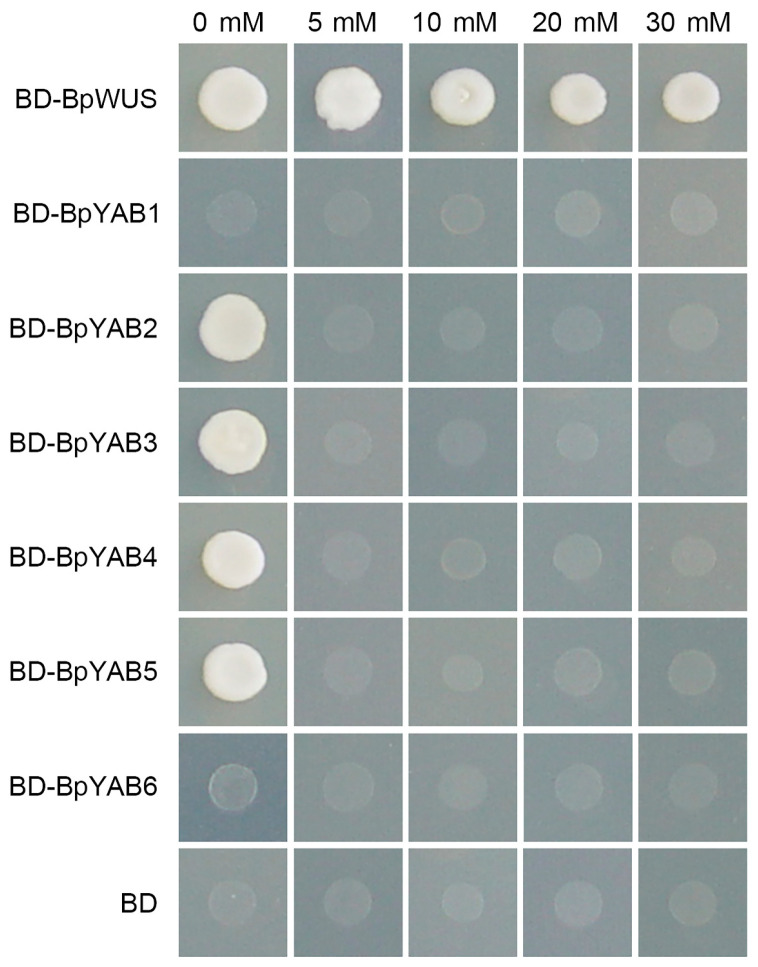
The transactivation activity of YABBY proteins in paper mulberry. The full-length ORF of each *BpYAB* gene was fused with *p*Bridge and transformed into AH109 yeasts. Then AH109 yeasts were selected from the SD-Trp-His medium. The *p*Bridge-BpWUS was used as a positive control, and the empty vector *p*Bridge (BD) was used as a negative control.

The transactivation activity of each BpYAB protein was tested by a yeast one-hybrid system in the synthetic dropout nutrient medium-Trp-His (SD-Trp-His) growth condition. The transactivation activity was characterized by the growth ability of the transformed yeast on the SD plate containing 0–30 mM 3-AT (3-Aminotriazole). The *p*Bridge-BpWUS was used as a positive control, and the pBridge (BD) was taken as a negative control [28]. The results indicated that four BpYAB proteins—BpYAB2, BpYAB3, BpYAB4, and BpYAB5—have transcriptional activity, while BpYAB1 and BpYAB6 did not exhibit transcriptional activity (Figure 2). None of the YABBYs in paper mulberry showed high-level transcriptional activity, as they could not grow on the selective medium containing 5–30 mM 3-AT.

**Figure 3:**
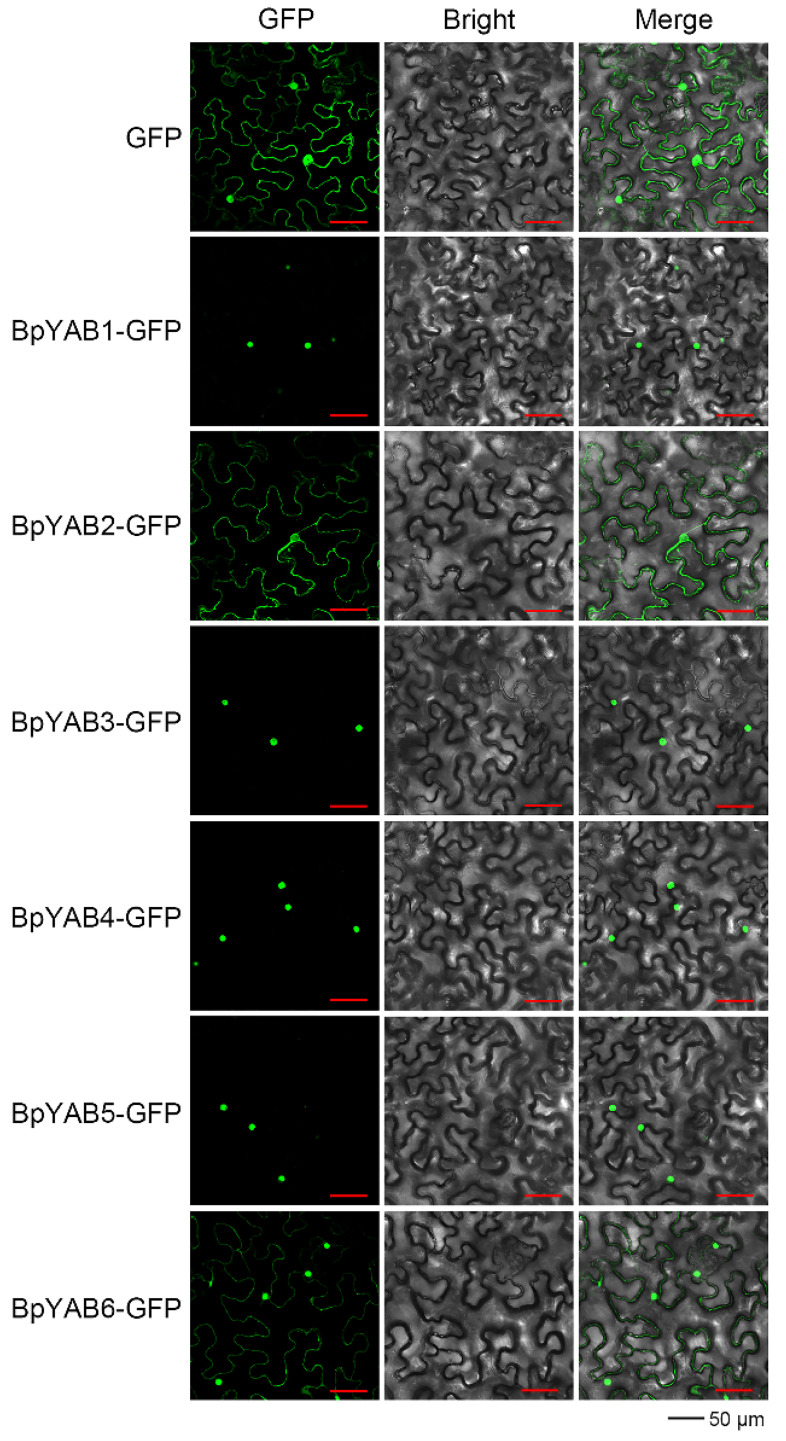
Subcellular localization of YABBY proteins. Transient expression of *p*CAMBIA1300-BpYABs-GFP fusion protein was performed in tobacco epidermal cells. *p*CAMBIA1300-GFP was used as a control. From left to right, the images show the green fluorescent field, bright field, and overlap of two illuminations. Bar = 50 µm.

The *p*CAMBIA1300-GFP was used as a positive control to illustrate the subcellular localization of BpYABs. The GFP signal of four BpYABs (BpYAB1, BpYAB3, BpYAB4, and BpYAB5) GFP fusion proteins was observed exclusively in the nucleus, suggesting that these four BpYABs were localized in the nucleus. The GFP signal of the BpYAB2-GFP and BpYAB6-GFP fusion proteins was in both the nucleus and cytoplasm (Figure 3), indicating that these two BpYABs were not unique nuclear localization proteins.

### 2.6. Expression Patterns of BpYAB Genes in Different Tissues

**Figure 4:**
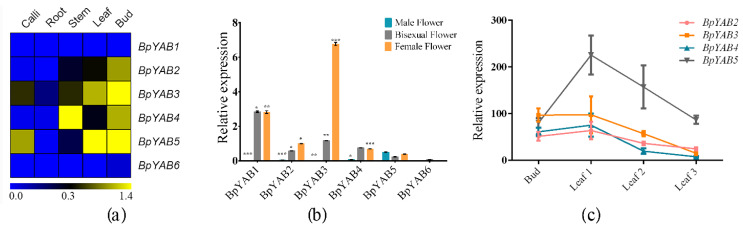
The expression patterns of *BpYAB* genes in different tissues. (**a**) The expression pattern of *BpYAB* genes in the callus, leaf, bud, stem, and root at the transcription level. (**b**) The expression levels of *BpYAB* genes in the female flower, male flower, and bisexual flower. * means *p* less than 0.05, ** represents *p* < 0.01 and *** represent *p* < 0. 001.Transcript levels of (**a**) and (**b**) were determined by qRT-PCR with three independent biological replications, and the expression of *BpGAPDH* was used as an internal control. (**c**) The expression levels (values of FPKM) of four *BpYAB* genes in the apical bud and leaf at three developmental stages. Leaf 1 is the newly grown leaf, visible to the human eye; Leaf 2 is the tender leaf; and Leaf 3 is the mature leaf. The data were obtained from the transcriptome of the paper mulberry.

None of the *BpYAB* members were expressed in the root, but were mainly expressed in the bud, stem, leaf and flower (Figure 4a), indicating that the *YABBY* family functioned during aboveground organ development. However, neither *BpYAB1* nor *BpYAB6* was expressed in the vegetative organs, including the bud, stem, and leaf (Figure 4a). This expression pattern was consistent with their counterparts in Arabidopsis, *CRC*, and *INO*, which were not expressed in vegetative organs as well. Except for the two abovementioned genes, the remaining four *BpYABs* were highly expressed in the bud (Figure 4a). Moreover, *BpYAB3* and *BpYAB5* were strongly expressed in the mature leaf and lowly expressed in the stem, implying that these two genes may be involved in leaf development. On the other hand, *BpYAB4* had a relatively higher expression in the stem and lower expression in the leaf, indicating that *BpYAB4* may function in stem growth (Figure 4a). Except for *BpYAB6*, the *BpYABs* were all expressed in the flowers, especially in the female flower and bisexual flower (Figure 4b). *BpYAB3* showed a higher expression in the female flower than in the bisexual flower (*p* < 0.001) among the rest of the *BpYABs*, and this implied that *BpYAB3* was specific for female flower development (Figure 4b). In addition, only three members were expressed in the male flower, and *BpYAB5* showed a higher expression than *BpYAB2* and *BpYAB4* (Figure 4b). To check the expression dynamics of four vegetative *BpYAB* genes in leaves, we obtained the FPKM values of three developmental stage leaves from the transcriptome sequencing data of paper mulberry. Leaf 1 means the newly grown leaf visible to the human eye, Leaf 2 is the tender leaf, and Leaf 3 represents the mature leaf. All the *BpYAB* genes showed a medium level in the bud and reached the highest expression in the Leaf 1 stage, then decreased with the leaf growing. The expression levels of *BpYAB5* were twice as high as those of the other three genes during leaf development (Figure 4c). It was interesting to find that *BpYAB5* showed high activity in many tissues, including both vegetative organs and reproductive organs, but mostly in leaves (Figure 4).

### 2.7. BpYAB3 and BpYAB5 Do Not Show a Polarity Distribution in the Leaf

**Figure 5:**
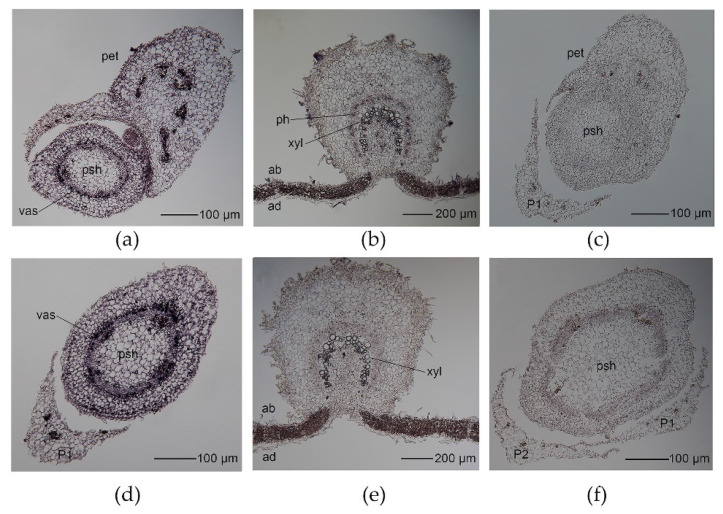
Expression patterns of *BpYAB3* and *BpYAB5* in the SAM and mature leaf by in situ hybridization. (**a**,**b**) Expression patterns of *BpYAB3* in the shoot apical meristem and mature leaf. (**c**) Sense probe for *BpYAB3* as a negative control. (**d**,**e**) Expression patterns of *BpYAB5* in the shoot apical meristem and mature leaf. (**f**) Sense probe for *BpYAB5* as a negative control. Abbreviation: ab, abaxial; ad, adaxial; pet, petiole; psh, primary shoot; p, primordium; ph, phloem; vas, vascular bundle; xyl, xylem.

*YABBY* genes are expressed in a polar pattern and function in promoting abaxial polarity in Arabidopsis, including leaf, flower and ovule. However, *YABBY* genes do not show a polar pattern in rice. To check whether the *YABBY* genes of the paper mulberry showed polar distribution in leaves, we conducted the RNA in situ hybridization analyses of *BpYAB3* and *BpYAB5* in the apical bud and mature leaf because these two genes were highly expressed in the bud and leaf. The results showed that both were expressed in the vascular bundle of primary shoot and leaf primordium, including xylem and phloem (Figure 5a,d). In the mature leaf, *BpYAB3* and *BpYAB5* were both expressed throughout the entire leaf blade and did not show a polar distribution (Figure 5b,e). Both *BpYAB3* and *BpYAB5* were expressed in the xylem of the mature leaf midrib, and *BpYAB3* was also expressed in the phloem of the mature leaf midrib. A negative control of the sense probe for *BpYAB3* and *BpYAB5* was used (Figure 5c,f). Overall, both *BpYAB3* and *BpYAB5* were expressed not only in the adaxial or abaxial lamina, specifically, but throughout the entire leaf blade, demonstrating that YABBYs in the paper mulberry may play a role in initiating outgrowth of the lamina.

### 2.8. Ectopic Expression of BpYABs Affects Arabidopsis Leaf Development

**Figure 6:**
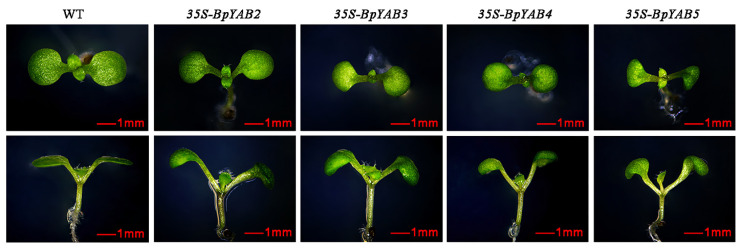
Effects on the morphology of the cotyledon in *A. thaliana* by ectopic overexpression of *BpYAB2*, *BpYAB3*, *BpYAB4*, and *BpYAB5*. The first row of photos shows the top view, and the second row of photos shows the side view.

Ectopic overexpression of *BpYAB2*, *BpYAB3*, *BpYAB4*, and *BpYAB5* in Arabidopsis affected the morphology of Arabidopsis leaves. Compared with the wild-type Arabidopsis (Col-0), the transgenic line showed that the cotyledons curled downward to form an inverted bowl-shaped leaf, and the leaf area was relatively smaller. The leaves of the wild-type Arabidopsis were flat (Figure 6). When the seedlings were transplanted to nutrient soil and cultured for 10 days, it was found that in the transgenic line, the true leaves of Arabidopsis also appeared curled at the leaf edge, and some of them showed a twisted phenotype. The growth of the entire plant was slower than that of the wild-type (Figure S5).

**Figure 7:**
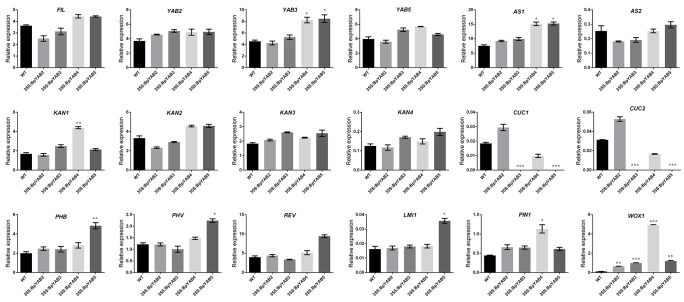
Expression of leaf-development-related genes in *A. thaliana* by ectopic expression of *BpYAB2*, *BpYAB3*, *BpYAB4*, and *BpYAB5*. * means *p* less than 0.05, ** represents *p* < 0.01 and *** represent *p* < 0.001. All the gene IDs and primers are listed in Table S5.

The ectopic overexpression of the *BpYAB2*, *3*–*5* genes in Arabidopsis did not affect the expression of the *AtYAB* genes (*YAB2*, *YAB3*, *FIL*, and *YAB5*) in Arabidopsis (Figure 7). Expression levels of *KAN2*, *KAN3* and *KAN4* genes functioning on the abaxial surface of the leaves, and of the genes *PHB* and REV specifically expressed in the paraxial plane, also did not change significantly. There was no difference in the related factors that controlled the development of the adaxial–abaxial axis in Arabidopsis (*AS1* and *AS2*). These results suggested that the ectopic overexpression in Arabidopsis did not cause the loss of the dorsoventral polarity. The expression of the *CUC* gene that controls the leaf margin development was significantly decreased in the 35S:BpYAB3 and 35S:BpYAB5 strains (*p* < 0.001), but slightly increased in the 35S:BpYAB2 strain. The expression of the downstream regulatory gene *WOX1* of YAB increased significantly in all the transgenic strains, especially in 35S:BpYAB4, increasing 48 times that in the WT. In addition, the expression of auxin polar transport *PIN1* doubled in the 35S:BpYAB4 strain (*p* < 0.05).

Therefore, we speculated that the abnormal leaf development caused by the ectopic expression of *BpYAB*s may be due to the fact that BpYABs promote the expression of their downstream gene WOX1 and involve changes in the distribution of auxin, although the influence differed in different ectopic expressions of *BpYAB* strains.

### 2.9. Ectopic Expression of BpYABs Affects Arabidopsis Silique Length and Seed Size

**Figure 8:**
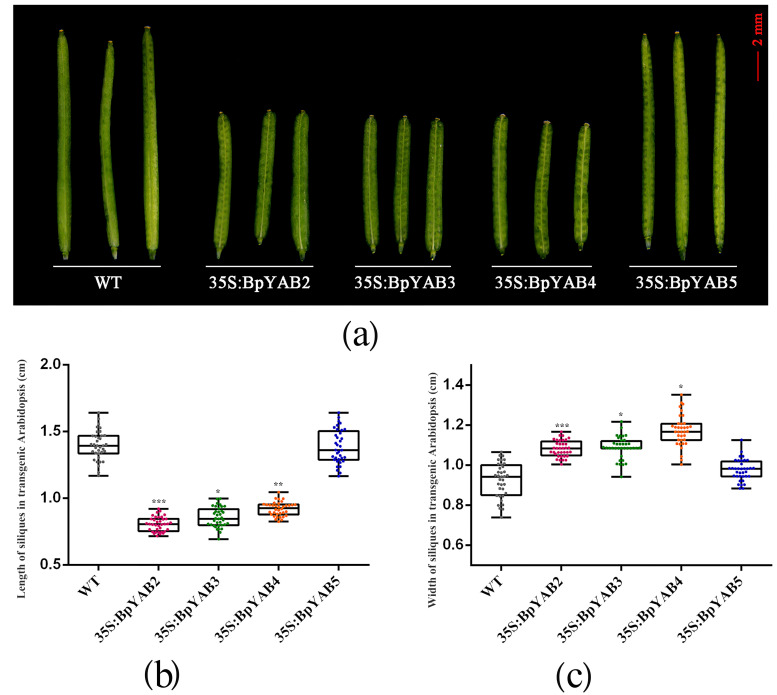
Effects on siliques in *A. thaliana* by ectopic overexpression of *BpYAB2*, *BpYAB3*, *BpYAB4*, and *BpYAB5*. (**a**) Morphology of siliques in *A. thaliana*. (**b**) Length of siliques in *A. thaliana*. (**c**) Width of siliques in *A. thaliana*. * means *p* less than 0.05, ** represents *p* < 0.01 and *** represent *p* < 0.001.

The ectopic expression of *BpYAB2*, *BpYAB3*, and *BpYAB4* will cause the Arabidopsis silique length to be significantly shorter (Figure 8). The length and width of the wild-type and transgenic Arabidopsis siliques were measured, and the results of statistical analysis of differences found that the lengths in the transgenic of *BpYAB2* (*p* < 0.001), *BpYAB3* (*p* < 0.05) and *BpYAB4* (*p* < 0.01) line were significantly shorter than those of the wild-type and 35S:*BpYAB5* lines (Figure 8a,b), and the widths were slightly greater than those of the wild-type and 35S:*BpYAB5* lines (Figure 8c). Therefore, the ectopic expression of *BpYAB2*, *BpYAB3*, and *BpYAB4* can cause the length of the Arabidopsis silique to be significantly shorter.

Additionally, the number of seeds in a single pod was counted. The results showed that the number of seeds did not decrease in all the transgenic plants (Figure S6a). Furthermore, observation and measurement of the sizes of mature seeds (Figure S7) showed that the length and width of seeds in the *BpYAB2*, *BpYAB3*, and *BpYAB4* strains were smaller than those of the wild-type and 35S:*BpYAB5* lines (Figure S6b). Moreover, the ectopic expression of *BpYAB2*, *BpYAB3* and *BpYAB4* can shorten the silique and seeds of Arabidopsis more than that of WT or 35S:YAB5, but does not affect the number of seeds in a single silique.

## 3. Discussion

Members of YABBY are seed-plant-specific transcription factors and function in lateral organ development. With the availability of the whole genome, YAB families have been identified in Arabidopsis, rice, tomato, soybean, and cotton [3,29,30]. However, few studies have been performed on *YABBY* genes in trees.

### 3.1. The Sequence Structures of YABBY Transcription Factors in Paper Mulberry Are Conserved

In this study, six *YABBY* genes were identified from the paper mulberry genome (Figure 1 and Table 1), and subsequent analysis showed that the structure of the *YABBYs* was relatively conserved among different plants. *BpYAB2* and *BpYAB6* had six exons, while the remaining four *BpYAB* genes had seven exons in the genomic DNA sequence (Figure S2a). The lengths of exon 4 and exon 5 were the same in the six *YABBY* members of paper mulberry: 49 and 76 bp, respectively. These two exons were conserved in different plants, possibly because they code the YABBY domain. Previous studies have shown that the core element of the C_2_C_2_-type zinc-finger domain with Zn^2+^ is the C-X_2_-C-X_9_-P-X_11_-C-X_2_-C motif, and this structure has also been found in the YABBY protein sequences of paper mulberry (Figure S2c). The putative helix-loop-helix “YABBY” domain has been reported to have a sequence similar to the first two helices of the high mobility group (HMG) box [31], and we found that BpYABs also contain typical helices of HMG boxes (Figure S2d), suggesting that the structure of the YABBYs is conserved among seed plants. Except for two conserved domains, many conserved motifs found in variable regions from each sub-family also showed similarities. Three motifs (motif 3, motif 4, and motif 9) were found in the C-terminal of the YABBY protein. Motif 3 was found in the vegetative clades, including *FIL*-like, *YAB2*-like, and *YAB5*-like clades, while motif 4 and motif 9 were only specific to the *FIL*-like clade (Figure S3b). Five motifs, located in the variable region between the two conserved domains (C_2_C_2_ zinc-finger and YABBY), were found only in *M. notabilis* and paper mulberry, but not in *A. thaliana* (Figure S3). Paper mulberry and *M. notabilis*, which both belong to the Moraceae family, shared the same conserved motif in the YABBY protein sequence, suggesting they may have a very close evolutionary relationship and may have evolved from a common ancestor.

### 3.2. Three BpYAB Proteins Are Typical Transcription Factors

The YABBY acts as a transcription factor, including a typical DNA binding site (YABBY domain) and an oligomerization site (C_2_C_2_ zinc-finger domain) [32]. We studied the subcellular localization and transactivation activity of the BpYAB family for the first time in a comprehensive way. Three BpYABs (BpYAB3, BpYAB4, and BpYAB5) showed typical characteristics of the transcription factor, including the transactivation activity and nuclear localization signal, as well. These results were consistent with VpYAB1 in grapes and BraYAB1 in cabbages, which belong to the *FIL*-like clade, as well as BpYAB3 and BpYAB4 [8,10]. MsYAB5 is the homologous gene of BpYAB5 in spearmint; both of them are in the *YAB5*-like clade and localized in the nucleus [11]. BpYAB2 could not localize in the nucleus specifically but could activate the downstream gene expression, implying that it needs to form complexes with other proteins to function in the nucleus. VpYAB2 and IaYAB2, which are members of the *YAB2*-like clade and are localized in the nucleus [8,9], differed from BpYAB2 in the paper mulberry, implying the diverse characteristics of YABBYs in different species. Additionally, BpYAB1 is a nuclear localization protein without the transactivation activity, implying that it might function as a transcriptional repressor. This result was in accordance with the *CRC* gene in Arabidopsis, which is localized in the nucleus and acts as a transcriptional repressor in the floral meristem [2]. Notably, BpYAB6 had neither the nuclear localization signal nor the transactivation activity, and the expression level was very low in the tissues detected, inferring that its function may not be as important as others in the BpYAB family.

### 3.3. YAB Family Underwent Expansion in the FIL-like Clade

The *YABBY* genes from seed plants can be divided into five groups: the *CRC*-like, *INO*-like, *FIL*-like, *YAB2*-like, and *YAB5*-like groups [12]. Members of YABBYs from 17 species, including 127 protein sequences, were evenly divided into these five groups. The resulting phylogenetic tree of dicotyledons showed that *YAB2*-like and *YAB5*-like clades had a close relationship, while *CRC*-like and *INO*-like clades might have originated from a common ancestor (Figure 1). In addition, the five YABBYs from the base angiosperm *A. trichopoda* were found in these exact five groups. These results indicated that YABBYs may diverge from a common ancestor within the same group, and the biological function of each group might be similar in different species. The *INO*-like and *CRC*-like clades contained 18 and 19 *YABBY* genes among the 17 species, and each group contained at least one gene from the two groups. One or two *YABBY* genes were found in the *YAB2*-like and *YAB5*-like clades, the number of *YABBY* genes in these two groups were 23 and 28, respectively. There were five more members in the *YAB5*-like clade than in the *YAB2*-like clade, and this may have resulted from the expansion of *GhYABs* in the *YAB5*-like clade (Figure 1). However, the number of YABBY genes in the *FIL*-like group, which was 39, was twice more than that in the *CRC*-like and *INO*-like groups. There was only one *YABBY* gene in the *FIL*-like clade from the base species of angiosperm *A. trichopoda*, but at least two members in other dicotyledon plants from Vitaceae to Solanaceae, indicating that there was an expansion event of *YABBY* in the *FIL*-like clade. We also found that *WOX* genes in the WUS clade underwent an expansion event in the paper mulberry as well, demonstrating that the expansion event is common in the transcription factor family in the paper mulberry.

### 3.4. The Function of BpYAB Genes Was Related to the Evolutionary Relationship

In Arabidopsis and other species, expressions of *CRC*-like and *INO*-like groups are restricted to reproductive organs, such as the carpel and ovule [14,32]. In contrast, members of the *FIL*-like, *YAB2*-like, and *YAB5*-like groups are mostly expressed in the leaves and are termed the “vegetative YABBYs” [20,33]. The vegetative angiosperm *YABBY* genes are exclusively expressed in leaf-homologous organs, including the leaf and flower. Evidence has shown that vegetative YABBYs act in several aspects of leaf development [34,35]. We identified six *YABBY* genes in the paper mulberry, and the number of YABBY families in the paper mulberry was the same as in the model plant, Arabidopsis. *YABBY* genes in Arabidopsis and paper mulberry can have a one-to-one correspondence in the phylogenetic tree, and their biological function may be similar, as well. The expression of *BpYAB* family members was not detected in the root, suggesting they were mainly expressed in aboveground organs such as the bud, leaf, stem, and flower. *BpYAB1* was only expressed in the female flower and bisexual flower in paper mulberry but was not expressed in the male flower or other vegetative organs (Figure 4a,b). This was consistent with its counterpart *CRC* gene in Arabidopsis, which functions in carpel development [14]. *BpYAB6*, the counterpart of the *INO* gene in Arabidopsis, was not expressed in the organs detected (Figure 4a,b), inferring that it may participate in other types of organ development that we did not detect in the paper mulberry. The remaining four *BpYAB* genes, belonging to the *FIL*-like, *YAB2*-like, and *YAB5*-like clades, were expressed in both vegetative and reproductive organs (Figure 4), and their functions were in line with corresponding *AtYAB* genes [19,36]. *BpYAB3* and *BpYAB5* were especially highly expressed in the bud and leaf (Figure 4a), suggesting they are significant to leaf formation. In addition, the four *BpYAB* genes played a meaningful role in the leaf development process, as well (Figure 4c). *BpYAB5* showed a high activity in all tissues, including the male flower and leaf, revealing that this gene is fundamental to the growth of paper mulberry. Altogether, the functions of the *BpYAB* gene are related to its evolution, demonstrating the conserved biological role in dicotyledons.

### 3.5. BpYAB Did Not Show the Adaxial–Abaxial Polarity Distribution in Lamina

It has been reported that *YABBY* genes show the abaxial expression pattern in the Arabidopsis leaf and the adaxial expression pattern in the maize leaf [1,23]. However, *YABBYs* in rice are fully expressed in the spikelet and midrib in the leaf, which do not show the polarity pattern. These results indicate that the polarity expression pattern may diverge in different species. The in-situ results of *BpYAB3* and *BpYAB5* showed that they were expressed in the vascular bundle of the primary shoot and leaf primordium and were not expressed in the lamina of the leaf primordium. In the mature leaf, *BpYAB3* and *BpYAB5* were both expressed throughout the leaf blade and did not show a polar distribution (Figure 5). *BpYAB3* was expressed in both the xylem and phloem of the midrib in the mature leaf, but *BpYAB5* was only expressed in the xylem, and this result was consistent with the expression levels of two *BpYAB* genes in the stem, where *BpYAB3* was higher than *BpYAB5* (Figure 4a). As mentioned earlier, the abaxial expression pattern may diverge in different species, which makes sense in paper mulberry, as well.

### 3.6. BpYABs Involved in Leaf and Silique Development

*AtCRC* specifically regulates carpel and nectary development [14]. According to the phylogenetic tree analysis, *BpYAB1* and *AtCRC* were homologous genes. Meanwhile, *BpYAB1* was only expressed in the female flowers but not in the vegetative organs of paper mulberry, which indicated that the genes of the CRC sub-clade mainly function in the development of floral organs, and their functions are relatively conservative. *BpYAB6* is homologous to *AtINO*, which is expressed in the outer integument of the ovule [37]. Sequence analysis revealed that BpYAB6 had no nuclear localization signal and lacked the ability to activate downstream reporter gene expression (Figure 2). *BpYAB6* was not expressed in the vegetative organs but only in the female flowers, and the expression level was very low. In this study, without the positive transgenic plants, it was not possible to speculate what growth and development processes *BpYAB6* and *BpYAB1* might perform in paper mulberry.

In Arabidopsis, FIL, YAB3, YAB2, and YAB5 have redundant functions and jointly regulate the formation of the abaxial surface of leaves. *BpYAB2* and *BpYAB5* are homologous with *AtYAB2* and *AtYAB5*, and the genes homologous to *AtFIL* are *BpYAB3* and *BpYAB4*. The expression patterns of these four *YAB* genes of Arabidopsis are similar, and all of them are specifically expressed in the abaxial face cells of lateral organs in the aboveground part [36]. The overexpression of *FIL* and *YAB3* genes in Arabidopsis can promote cell development at the distal end of leaves [1,19].

*OsYAB3*, *OsYAB4*, and *OsYAB5* are closely related to the *FIL*/*YAB3* of Arabidopsis. The expression of *OsYAB3* was detected in both the leaves and floral organs, and the RNAi transgenic rice leaves of OsYAB3 became twisted, knotted, or leafless ears, but this does not affect the polarity development of the leaves. At the same time, *YAB3*, *WOX3*, and *KNOX1* are involved in the formation of apical organs in rice [38]. The overexpression of OsYAB4 in Arabidopsis shows irregular leaf veins. It has been speculated that OsYAB4 is related to the development of rice vascular tissue [6]. *TOB1* (*OsYAB5*) is mainly involved in rice spikelet development [21]. OsYAB6 is closely related to OsYAB1 and belongs to the YAB2-like sub-clade. *OsYAB6* regulates the morphology of the paraxial end of the leaf by affecting the development of vesicle cells [39]. Except for *OsYAB7*, other rice *YABBY* genes are involved in the growth and development of the lateral organs, but they do not have a distribution pattern of dorsoventral polarity in the leaf.

The expression patterns of *BpYAB3* and *BpYAB5* in paper mulberry were similar, and both of them were highly expressed in mature leaves. Additionally, they were fully expressed in the lamina of paper mulberry without the adaxial–abaxial polarity. We speculated that *BpYAB* might play a role in the later development of leaves and affect the development of the leaf shape by regulating the expansion of the leaf blade. *BpYAB4* was highly expressed in the paper mulberry stems, but lower in the leaves. Furthermore, *BpYAB2*, *BpYAB3*, *BpYAB4*, and *BpYAB5* were all expressed in the leaf and bud, while *BpYAB3* and *BpYAB5* exhibited the higher level of expression (Figure 4a). The ectopic overexpression of *BpYAB2*, *BpYAB3*, *BpYAB4*, and *BpYAB5* caused the Arabidopsis leaves to become smaller and curl downwards. This phenotype was similar to that of Arabidopsis auxin mutant *axr1*, in which the lack of auxin causes the leaves to become smaller and curl downwards, but in *35S:BpYAB4*, the expression of the auxin polar transport factor *PIN1* increased, suggesting that the auxin content of *35S:BpYAB4* might be increased. Meanwhile, the expression pattern of *CUC1* and *CUC2* in the transgenic Arabidopsis was similar in that they showed slightly more upregulation in *35S:BpYAB2* than that in WT, and slightly more downregulation in *35S:BpYAB4* than that in WT, but almost undetectable expression in *35S:BpYAB3* and *35S:BpYAB5*. *CUC1* and *CUC2* are essential for shoot meristem initiation, as well as to promote carpel margin meristem formation during the Arabidopsis gynoecium development [40]. Furthermore, CUC1 and CUC2 can regulate cytokinin homeostasis to determine the ovule number in Arabidopsis [41]. Besides, previous studies and our experimental results showed that both an increase and decrease in auxin levels caused the leaves to curl downwards and shrink. Therefore, we speculated that the lack of auxin homeostasis led to the downward shape and curl shrinks of the leaves. In addition, the *YAB* gene might also indirectly participate in the auxin regulation pathway. The silique of the Arabidopsis *crc* mutant appeared to be shorter and thicker, and the tips could not be fused [14]. The Arabidopsis siliques were also found to be shorter and thicker in the *35S:BpYAB2*, *35S:BpYAB3*, and *35S:BpYAB4* ectopic expression lines (Figure 8), but the tip could be fused. The mature seeds were smaller than the wild-type (Figure S6), but the number did not change significantly, indicating that the abnormal expression of these *BpYABs* could also affect the morphology of the Arabidopsis silique.

Overall, the *YABBY* gene expression pattern in dicotyledonous plants is relatively conservative, mainly distributed at the abaxial end of the leaf, but it has other functions in different dicotyledonous plants. Although *YABBY* is defined as specifically expressed on the abaxial surface of the leaf, more and more studies have shown that YABBY also plays a role in the lateral growth of later leaves. In monocots, the function of *YABBY* genes is more diversified, mainly involved in the development of the floral organs. Thus, we speculated that YABBY has produced a functional differentiation in the evolution of dicotyledonous and monocots plants, not only limited to the abaxial cells of the organ development but presenting a diversified phenomenon.

## 4. Materials and Methods

### 4.1. Identification and Gene Sequence Analysis of the BpYAB in Paper Mulberry

To identify YABBY candidates in paper mulberry, we used protein sequences of *A. thaliana* and *M. notabilis* to search the paper mulberry protein database by employing the blastp. Then the blastn was used to identify potential genes in the paper mulberry genome [26]. The putative YABBY members were aligned with other YABBYs in *A. thaliana* and *M. notabilis* to check the conserved amino acid, and the predicted protein sequences lacking the C_2_C_2_ domain or YABBY domain were excluded. Finally, RACE-PCR was used to obtain and check the full length of *YAB* genes from paper mulberry.

The chromosomal positions and relative distances of the *BpYAB* genes were drawn with the online tool MG2C (Map Gene2 Chromosome V2) in Figure S1 (http://mg2c.iask.in/mg2c_v2.0, accessed on 20 August 2018). The amino acid sequences of putative YABBY proteins were submitted to the ProtParam of Expasy http://web.expasy.org/protparam, accessed on 5 August 2018) to calculate the molecular weights and theoretical isoelectric points in Table 1. The gene structure information of six *BpYAB* genes was obtained from the genome of the paper mulberry [26] and was drawn by a software package called Illustrator of Biological Sequences (IBS) [42] in Figure S2a. The conserved C_2_C_2_ domain and YABBY domain were aligned by MUSCLE in Geneious software and adjusted manually, and the relative positions in Figure S2b were drawn with IBS. For the analysis of *cis*-elements in the promoters of *BpYAB* genes, the upstream sequences (2 kb) from the translation start sites of the *BpYAB* genes were submitted to PLACE database (https://www.dna.affrc.go.jp/PLACE/, accessed on 5 January 2022) to identify the *cis*-acting regulatory elements in Table 2 and Table S4.

### 4.2. Phylogenetic Analysis and Conserved Motif Identification of BpYAB Proteins

The 127 YAB proteins of 17 plants were obtained from the PlantTFDB (Plant Transcription Factor Database) (http://planttfdb.cbi.pku.edu.cn/index.php, accessed on 21 June 2018). Evolutionary analyses were conducted in MEGA7, and the number of bootstrap replicates was 1000 [43]. The sequence information is provided in Table S1. The evolutionary history of YABs was inferred using the NJ (Neighbor-Joining) method. The optimal tree with the sum of the branch length was 19.12922157. The Poisson correction method was used to calculate the evolutionary distances. All ambiguous positions were removed for each sequence pair. The conserved motifs of YABBY proteins in *A. thaliana*, paper mulberry, and *M. notabilis* were identified using the motif elicitation tool MEME (http://meme.nbcr.net/meme/, accessed on 21 June 2018) with the following parameters: any number of repetitions, a maximum of 10 misfits with an optimum motif width from 6 to 200 amino acid residues.

### 4.3. Subcellular Localization and Transactivation Activity Assay of BpYAB Proteins

The open reading frame (ORF) of *BpYABs* was inserted into the *p*CAMBIA1300-GFP vector to generate the BpYAB-GFP fusion protein. The enzymes and primers used to construct the vectors are listed in Table S6. The *p*CAMBIA1300-BpYAB-GFP plasmid was transformed into *A. tumefaciens* (EHA105) and introduced into the *N. benthamiana* epidermal cell. After 48 h cultivation, fluorescence was examined by laser confocal microscopy (Leica TCS SP5, Wetzlar, Germany).

To perform the transactivation activity assay, the ORF of *BpYABs* was cloned into the *p*Bridge vector. The restriction enzymes and primers are provided in Table S7. The *p*Bridge-BpYABs vectors were transformed into *Saccharomyces cerevisiae* (AH109, BD Biosciences, Palo Alto, CA, USA) according to the manufacturer’s protocol. Transformed yeasts were screening cultured on SD medium without His and Trp. Then, the positive yeast cells were dropped on SD-Trp-His plates containing 3-AT (0 to 30 mM) and incubated at 30 °C for about 3 days. This experimental procedure was referenced in Tang et al. [28].

### 4.4. Plant Materials for qRT-PCR and RNA-Seq Data Analysis

To gain insight into the positions where the BpYAB genes were active, qRT-PCR was performed for different tissues, including the callus, bud, leaf, stem and root of tissue culture seedlings and the flowers of perennial paper mulberry from a wild field. For tissue specificity, plantlets, except the flower, were cultured on the MS culture media in an artificial climatic chamber at 26 °C for 14/10 h (day/night) for a month. Three kinds of paper mulberry flowers were obtained from three individual plants growing in the experimental field in the resource nursery, which belonged to our laboratory. All samples were frozen in liquid nitrogen and stored at −80 °C. Total RNAs of each sample were extracted with a kit (TransGen, Beijing, China). RNA quality and purity were evaluated with an OD260/280 ratio, and the value of RIN (RNA integrity number) was tested using the NanoDrop 2000 (Thermo Fisher, Waltham, MS, USA). First-strand cDNA synthesis was performed using PrimeScript RT Reagent Kit, (Takara, Dalian, China). Quantitative real-time PCR (qRT-PCR) was conducted on a real-time PCR system MX3000PTM, (Agilent Stratagene, Santa Clara, CA, USA). Each reaction was comprised of 20 µL, including 10 µL PCR Mix of SYBR-Green PrimeScript RT-PCR Kit (,Takara, Dalian, China), 2 µL template, 0.5 µL of each primer, 0.5 µL ROX, and 6.5 µL ddH_2_O. Three technical replicates were taken for each sample. The 2^−ΔΔCt^ method was used to analyze the expression levels of target genes, and the data were normalized by the transcript level of the *BpGAPDH* gene. The p value was calculated by means of the T student test and variance analysis (ANOVA) with significance levels of 5% (*p* < 0.05). All the primers for qRT-PCR are listed in Table S8. The FPKM values of four *BpYAB* genes (Table S9) were obtained from the RNA-seq data of the paper mulberry [26].

### 4.5. In Situ Hybridization of BpYAB3 and BpYAB5

The freshly collected apical bud and mature leaves were fixed immediately in FAA (3.7% formaldehyde, 5% acetic acid, and 50% ethanol) solution. The plant material was treated and detected as reported in [44]. The sequence-encoding C-terminal regions of *BpYAB3* (nucleotides 679–996 of ORF) and *BpYAB5* (nucleotide positions 517–819 of ORF) were amplified and transcribed as probes (Digoxigenin RNA labeling kit, Roche, Shanghai, China).

### 4.6. Agrobacterium-Mediated Transformation of Arabidopsis

The ORF regions of *BpYAB*2, *BpYAB*3, *BpYAB*4, and *BpYAB*5 were amplified using primers with restriction sites and ligated to the *p*CAMBIA1300 vector with T4 ligase (Takara, Dalian, China). The constructed vector was finally transferred into Agrobacterium GV3101. The Arabidopsis inflorescence was infected by the Agrobacterium for transformation, and the obtained seeds were screened with 50 μg/mL hygromycin B. Five overexpression lines for every *BpYAB* were obtained, and the leaf phenotype trend was consistent, so we selected one of them and the seedlings of T2 generation were used for further analysis.

### 4.7. The Morphological Observation of Transgenic Arabidopsis

The transgenic Arabidopsis was germinated in a Petri dish. On the 12th day, when the cotyledons unfolded and the true leaves were just about to grow out, the first phenotypic observation and photographs were taken. Then the seedlings were transplanted to the soil in the nutrient bowl. Observations and photographs were taken on the 8th, 10th, and 13th days after transplanting. During the grouting period, the length and width of the silique of each transgenic plant were measured, and there were more than 30 plants for each line. After the seeds matured, the number of seeds and the size of the seeds in a single silique were measured, and the total number in each line was more than 30. The p value was calculated by means of the T Student test and variance analysis (ANOVA) with significance levels of 5% (*p* < 0.05). The wild-type plants (WT) were used as controls.

### 4.8. The Statistical Analysis of Leaf Gene Expression in Arabidopsis

The expression level of genes involved in adaxial–abaxial leaf development were analyzed by qRT-PCR. The transcript levels were normalized against the expression of *AtACTIN2* (AT3G18780). Three biological replicates were taken in this experiment. The 2^−ΔΔCt^ method was used to analyze the data and the p value was calculated by means of the T Student test and variance analysis (ANOVA) with significance levels of 5% (*p* < 0.05). The GenBank accession numbers of Arabidopsis used in this study are listed as follows: *AtYAB2* (AT1G08465), *AtYAB3* (AT4G00180), *AtYAB5* (AT2G26580), *AtFIL* (AT2G45190), *AtAS1* (AT2G37630), *AtAS2* (AT1G65620), *AtKAN1* (AT5G16560), *AtKAN2* (AT1G32240), *AtKAN3* (AT4G17695), *AtKAN4* (AT5G42630), *AtCUC1* (AT3G15170), *AtCUC2* (AT5G53950), *AtPHB* (AT2G34710), *AtPHV* (AT1G30490), *AtREV* (AT5G60690), *AtLMI1* (AT5G03790), *AtPIN1* (AT1G73590), and *AtWOX1* (AT3G18010). Primers of qRT-PCR used in transgenic Arabidopsis are found in Table S5.

## 5. Conclusions

In this study, six *YABBY* genes were identified from paper mulberry and were evenly divided into five subfamilies from the phylogenetic analysis. Four members were located in chromosomes and the other two were in scaffolds. Subsequent sequence analysis revealed that *BpYAB* genes possessed six or seven exons and had several *cis*-elements that were development-, phytohormone-, and environment-related in the promotor region. Three members, *BpYAB3*, *BpYAB4*, and *BpYAB5*, were typical transcription factors, which were localized in the nucleus and possessed transactivation ability. Furthermore, *BpYAB* members were mainly expressed in aboveground organs, such as the leaf and flower. *BpYAB3* and *BpYAB5* showed full expression in the lamina of the mature leaf but did not show the adaxial or abaxial polarity in the paper mulberry as described in other species. Ectopic expression of four *BpYABs* caused leaves to curl downwards and shrink, and three of them affected the morphology of the Arabidopsis silique. Ultimately, our work sheds light on the evolutionary history and expression patterns of *BpYAB*s and provides fundamental information for understanding the precise function of YABBY in woody plants for further research.

## Figures and Tables

**Table 1:** Sequence information of the *BpYAB* family in paper mulberry.

Name	Gene ID	Chr.	Genomic Location	Direction	AA	*M*_W_(Da)	*p*I
*BpYAB1*	Bp0940004	scaffold094	52,363–54,474	-	180	20,070	9.32
*BpYAB2*	Bp09g0299	9	4,247,279–4,253,760	reverse	190	21,027	8.69
*BpYAB3*	Bp06g0033	6	408,527–411,063	reverse	222	24,558	7.77
*BpYAB4*	Bp01g1037	1	3,368,630–3,370,433	forward	226	25,048	9.04
*BpYAB5*	Bp1460001	scaffold146	32,046–36,003	-	194	21,896	8.77
*BpYAB6*	Bp07g0370	7	12,253,864–12,256,651	forward	179	20,222	4.97

Chr.: chromosome; AA: amino acid; *M*_W_: molecular weight; Da: dalton; *p*I: isoelectric point.

**Table 2:** Predicted *cis*-elements in *BpYAB* promoters.

Cis-Element		Function	*BpYAB1*	*BpYAB2*	*BpYAB3*	*BpYAB4*	*BpYAB5*	*BpYAB6*
Development	CCAAT-box	MYBHv1 binding site				◆		◆
	O2-site	zeatin metabolism regulation	◆	◆		◆		◆
	RY-element	seed-specific regulation			◆			
	MSA-like	cell cycle regulation						◆
Hormone	ABRE	abscisic acid responsiveness	◆		◆	◆		
	AuxRR-core	auxin responsiveness				◆		
	P-box	gibberellin-responsive	◆					
	TATC-box	gibberellin-responsiveness		◆		◆		◆
	TCA-element	salicylic acid responsiveness		◆			◆	
	TGA-element	auxin-responsive		◆				
Environment	ARE	the anaerobic induction		◆	◆	◆	◆	◆
	LTR	low-temperature responsiveness		◆				
	MBS	drought-inducibility	◆					◆
	WUN-motif	wound-responsive					◆	

◆ this cis-element was exsit in the promoter.

## Data Availability

Not applicable.

## Supplementary Materials

The following are available online at https://www.mdpi.com/article/10.3390/ijms23031670/s1.
