# Supplementary material for: Genome-Wide Analysis of BpYABs and Function Identification Involving in the Leaf and Silique Development in Transgenic Arabidopsis
Source: Int J Mol Sci. 2022 Jan 31;23(3):1670. doi: 10.3390/ijms23031670 (PMC8836020; doi:10.3390/ijms23031670)
Supplement: Supplementary file 1 [file ijms-23-01670-s001.zip › Caption of Supplementary files.pdf]

Figure S1: The chromosomal locations of YABBY genes in paper mulberry. The chromosome information of six BpYAB genes were obtained from the genome data of B. papyrifera. Four BpYABs are located in chromosomes and the other two were in the scaffolds.

Figure S2: The schematic of YABBY gene structure and conserved domain of YABBY protein in paper mulberry. **(a)** The gene structure of six BpYAB genes. Length of genomic sequences is displayed by black line, the exons are labeled with white-box, the nucleotide sequence coding C2C2 domain and YABBY domain are colored by red and green, respectively. Bar = 500 bp. **(b)** The length of amino acid of each BpYAB proteins is colored by gray, C2C2 domain and YABBY domain are colored by red and green as well. Bar = 30 aa. The amino acid sequences of C2C2 domain and YABBY domain are shown in Figure S2c and S2d. Letter B is Asx, representing Aspartic acid (Asp) and Asparagine (Asn). Letter X represents all amino acid. The conserved amino acids are marked with asterisks in Figure S2c and S2d.

Figure S3: Phylogenetic tree and conserved motifs of YABBYs among *A. thaliana*, *M. notabilis* and *B. papyrifera*. **(a)** The phylogenetic tree (NJ) was reconstructed by MAGE7 using 18 YABBY sequences from *A. thaliana*, *M. notabilis* and *B. papyrifera*. The *FIL*-like, *YAB2*-like, *YAB5*-like, *INO*-like, *CRC*-like are colored with sky-blue, green, red, pink and navy blue, respectively. **(b)** The length of YABBY proteins is represented by black line. Ten motifs were detected from the motif elicitation tool MEME. The motif 1 (green) is YABBY domain and motif 2 (yellow) is C_2C_2 domain. The conserved motifs among the members are highlighted in colored boxes with an arranged number, and the sequences of the motifs are listed in Figure S4. Bar = 30 aa.

Figure S4: The sequences of the motifs 1-10.

Figure S5: Effects on leaf development in *A. thaliana* by ectopic overexpression of *BpYAB2*, *BpYAB3*, *BpYAB4* and *BpYAB5*. From left to right, it suggested the morphology of leaf after 8-, 10- and 13-days growth in soil.

Figure S6: The statistics on morphology of seeds in *A. thaliana* through ectopic expression of *BpYAB4*. **(a)** The number of seeds per siliques. **(b)** The scatter plot of the length and the width of seeds in *A. thaliana*.

Figure S7: Observation and measurement of the sizes of mature seeds for transgenic Arabidopsis.

Table S1 Protein sequences were used to constructed the evolutionary tree.

Table S2 The restriction enzymes and primers used to construct the recombinant expression vectors for subcellular localization

Table S3 The restriction enzymes and primers used to construct the recombinant expression vectors for transactivation activity assay

Table S4 The primer sequences of *BpYABs* used in qRT-PCR

Table S5 The FPKM values of four *BpYAB* genes in bud and developmental leaves from RNA-seq data

Table S6 The primers used in the qRT-PCR in the transgenic plants

Table S7 Sequence information of BpYAB family in paper mulberry

Table S8 The length of exons from *BpYAB* genomic sequences

Table S9 The sequence and function of predicted cis-elements in promoter of *BpYABs*
