# Supplementary figures and images for: Genome-Wide Analysis of BpYABs and Function Identification Involving in the Leaf and Silique Development in Transgenic Arabidopsis
Source: Int J Mol Sci. 2022 Jan 31;23(3):1670. doi: 10.3390/ijms23031670 (PMC8836020; doi:10.3390/ijms23031670)

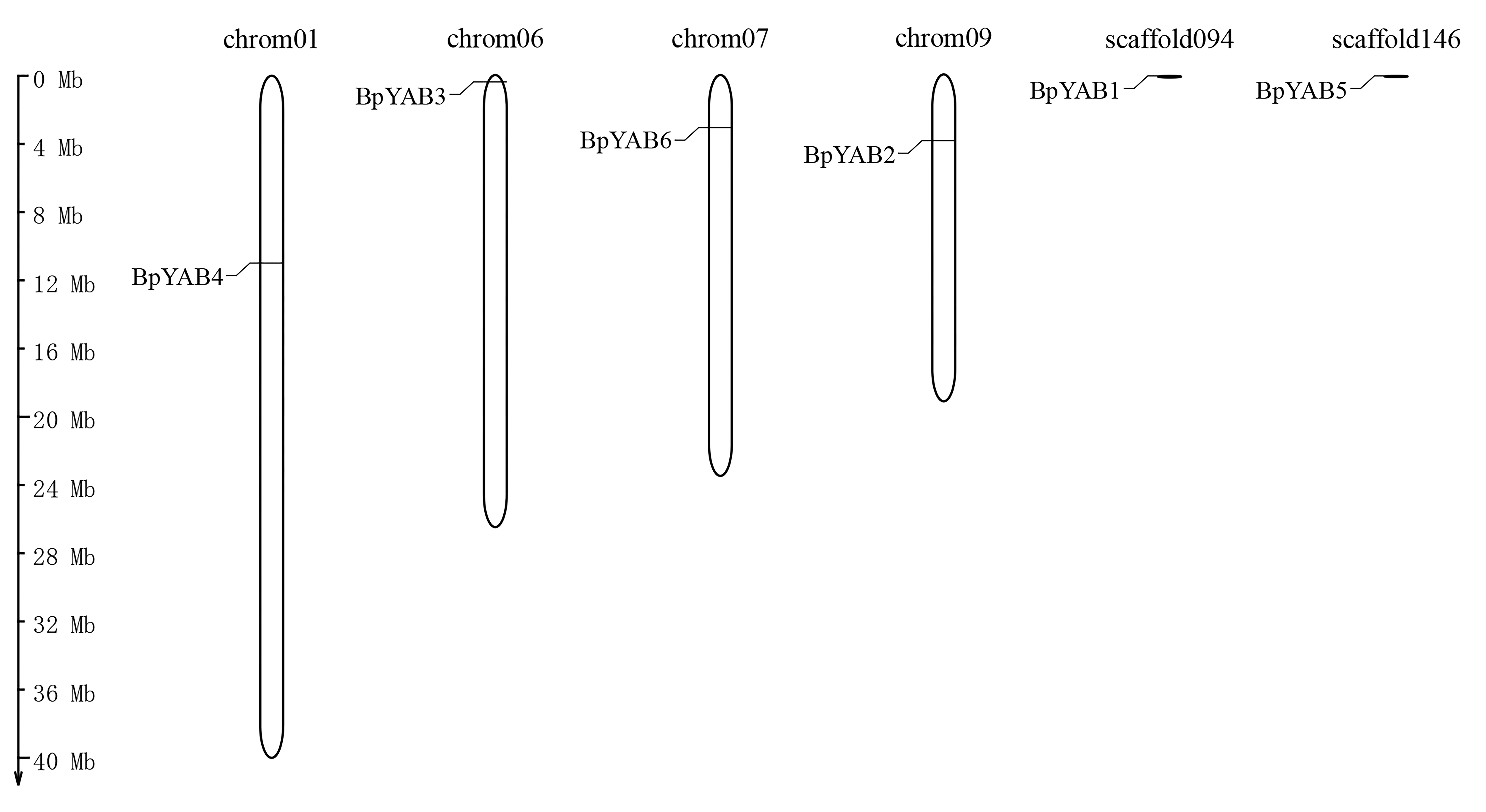

Supplement: Supplementary file 1 [file ijms-23-01670-s001.zip › Fig. S1.tif]

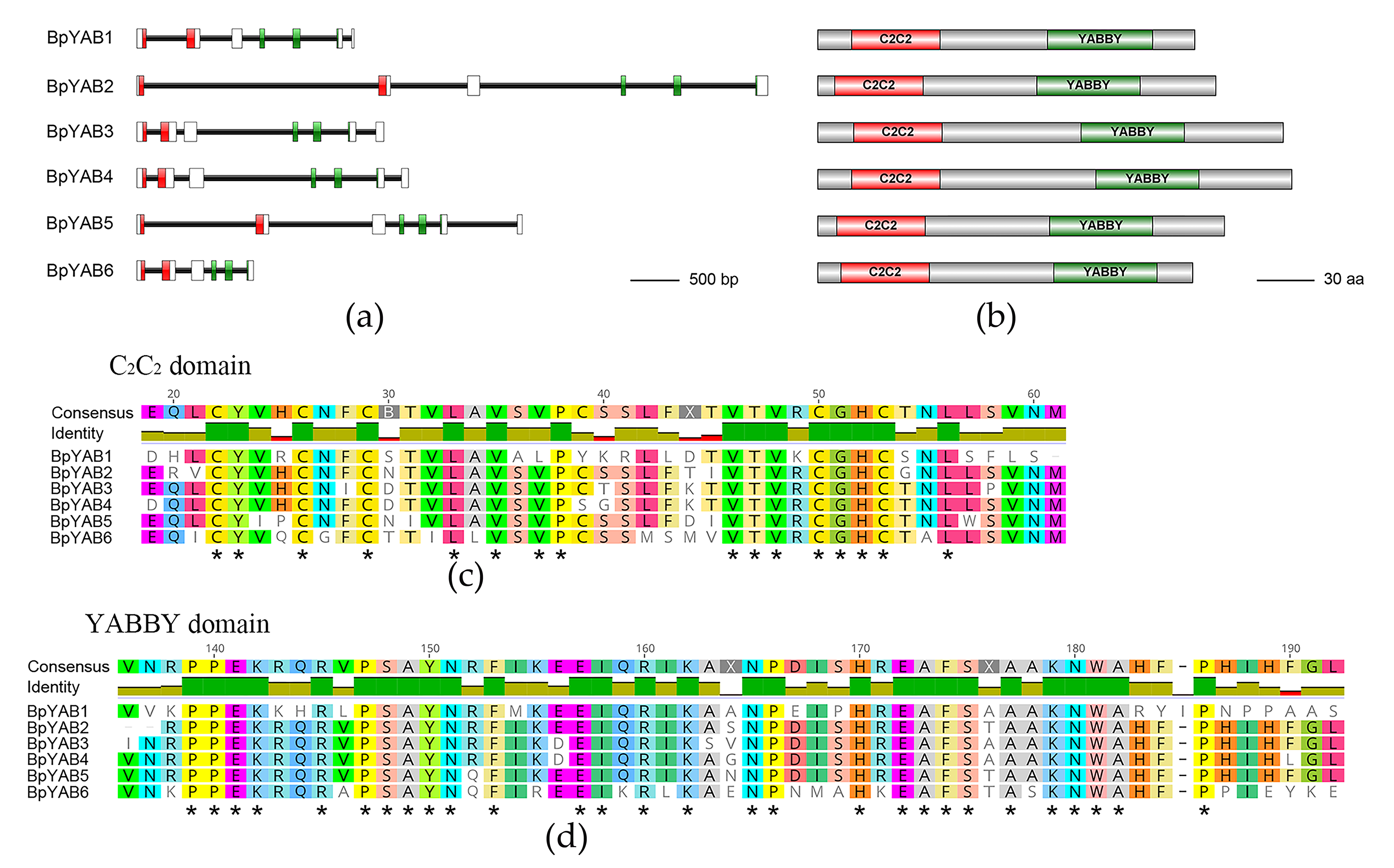

Supplement: Supplementary file 1 [file ijms-23-01670-s001.zip › Fig. S2.tif]

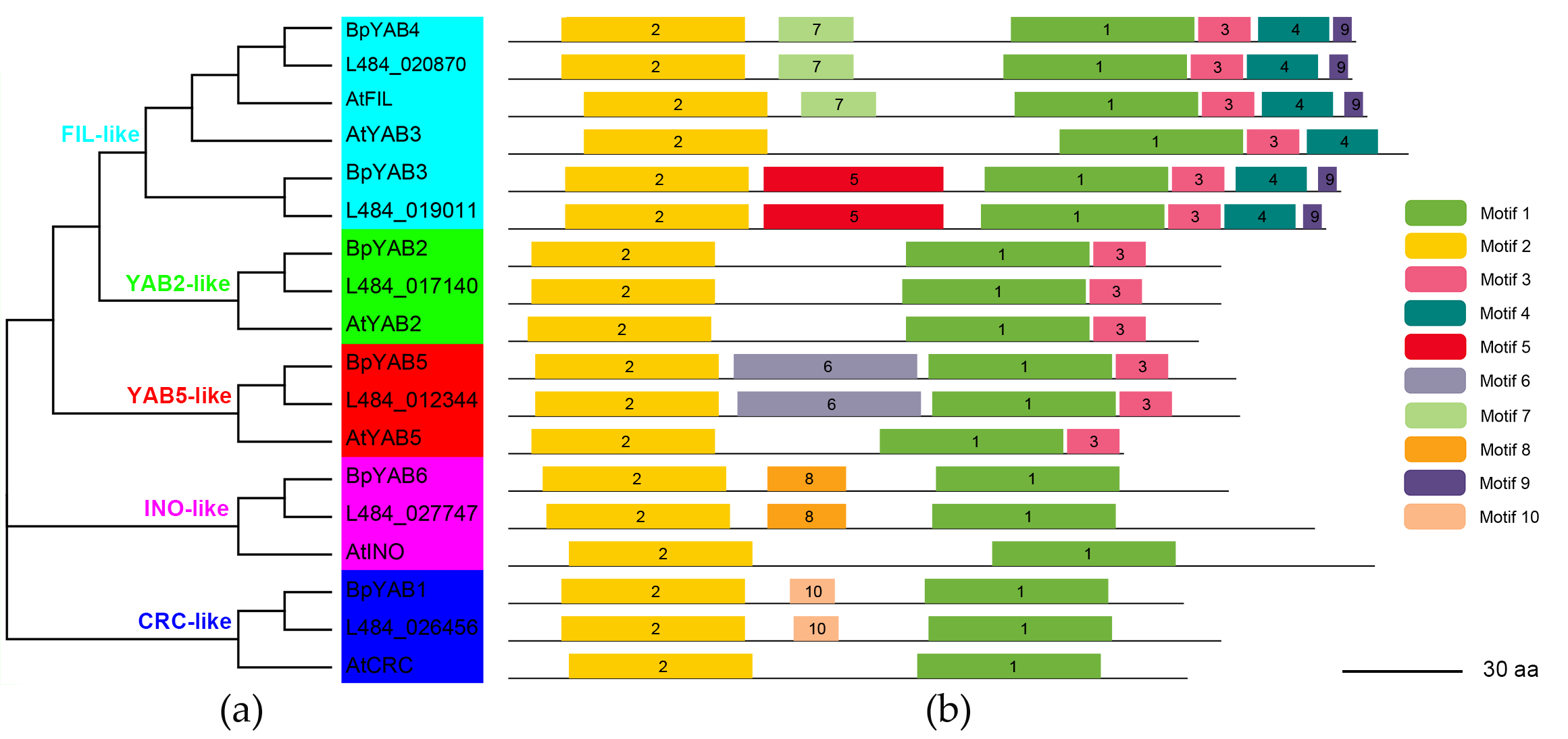

Supplement: Supplementary file 1 [file ijms-23-01670-s001.zip › Fig. S3.tif]

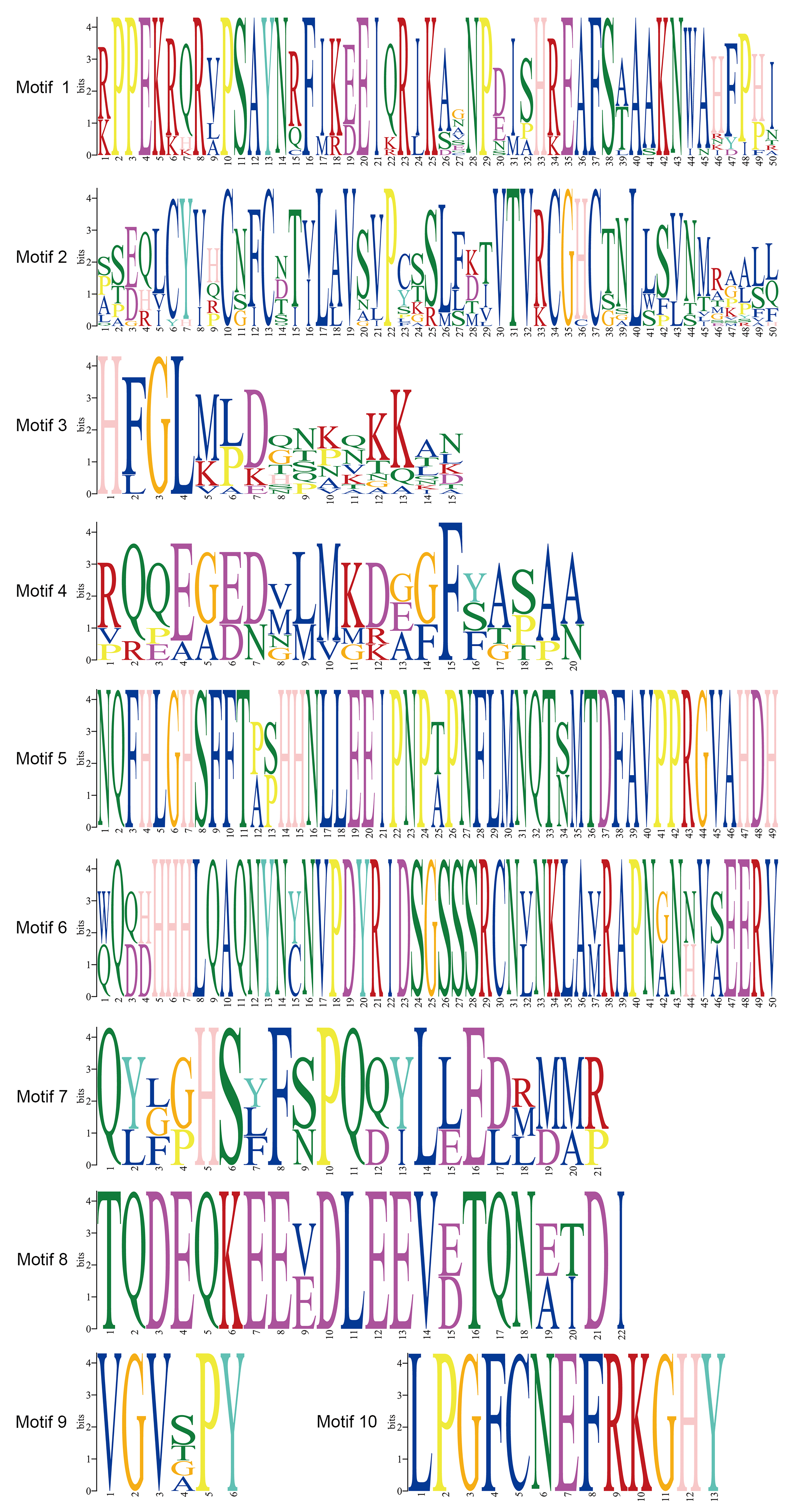

Supplement: Supplementary file 1 [file ijms-23-01670-s001.zip › Fig. S4.tif]

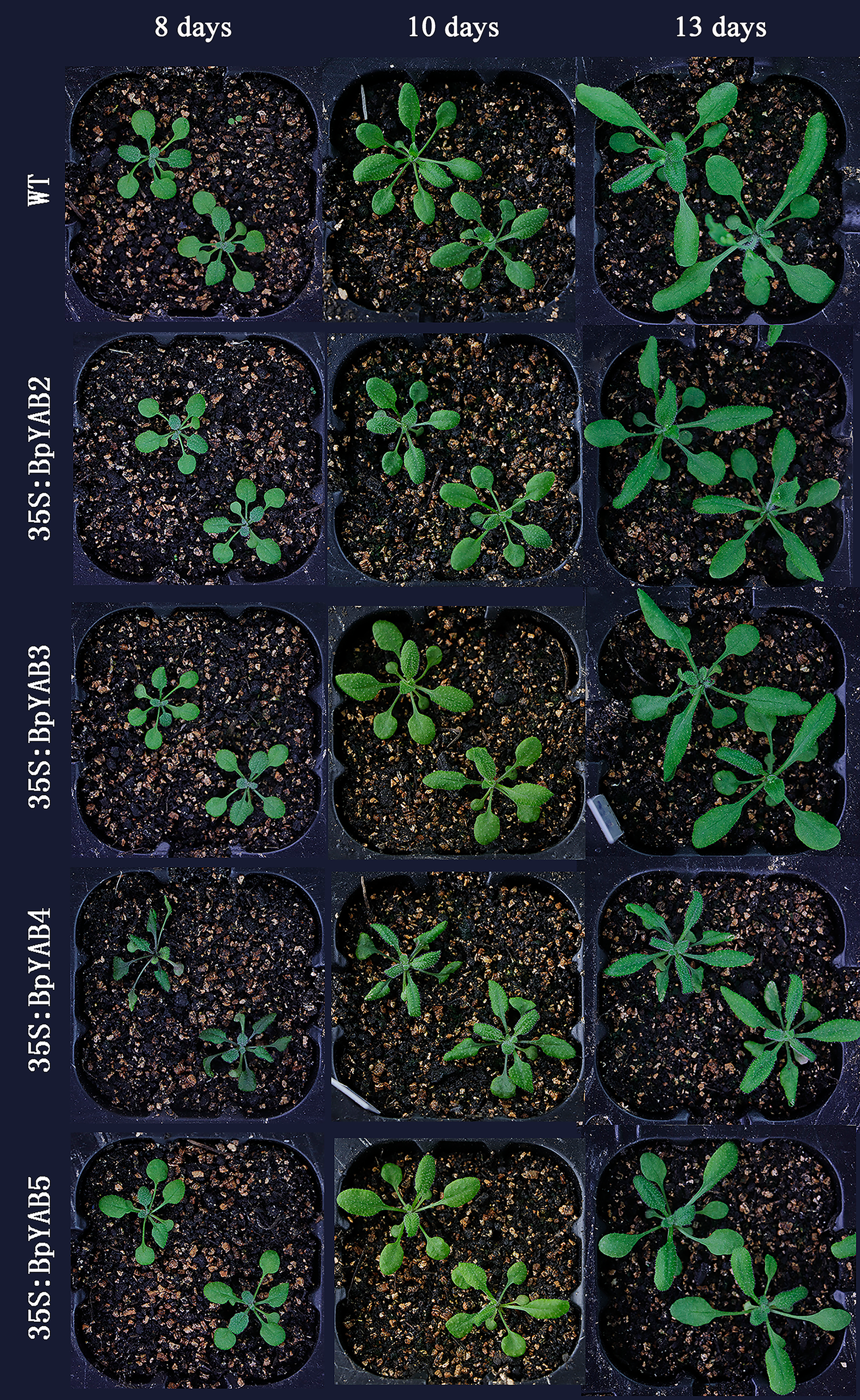

Supplement: Supplementary file 1 [file ijms-23-01670-s001.zip › Fig. S5.tif]

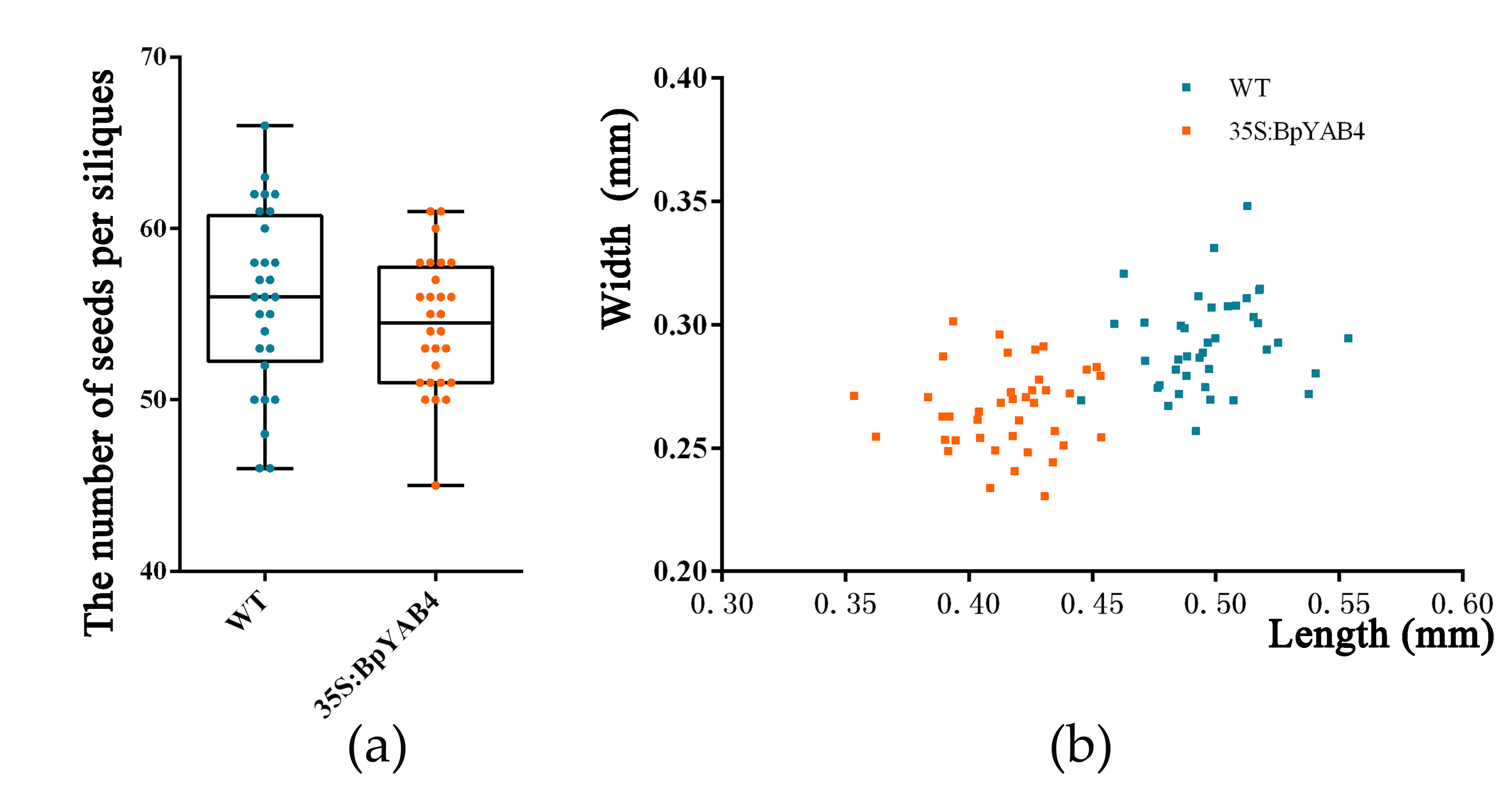

Supplement: Supplementary file 1 [file ijms-23-01670-s001.zip › Fig. S6.tif]

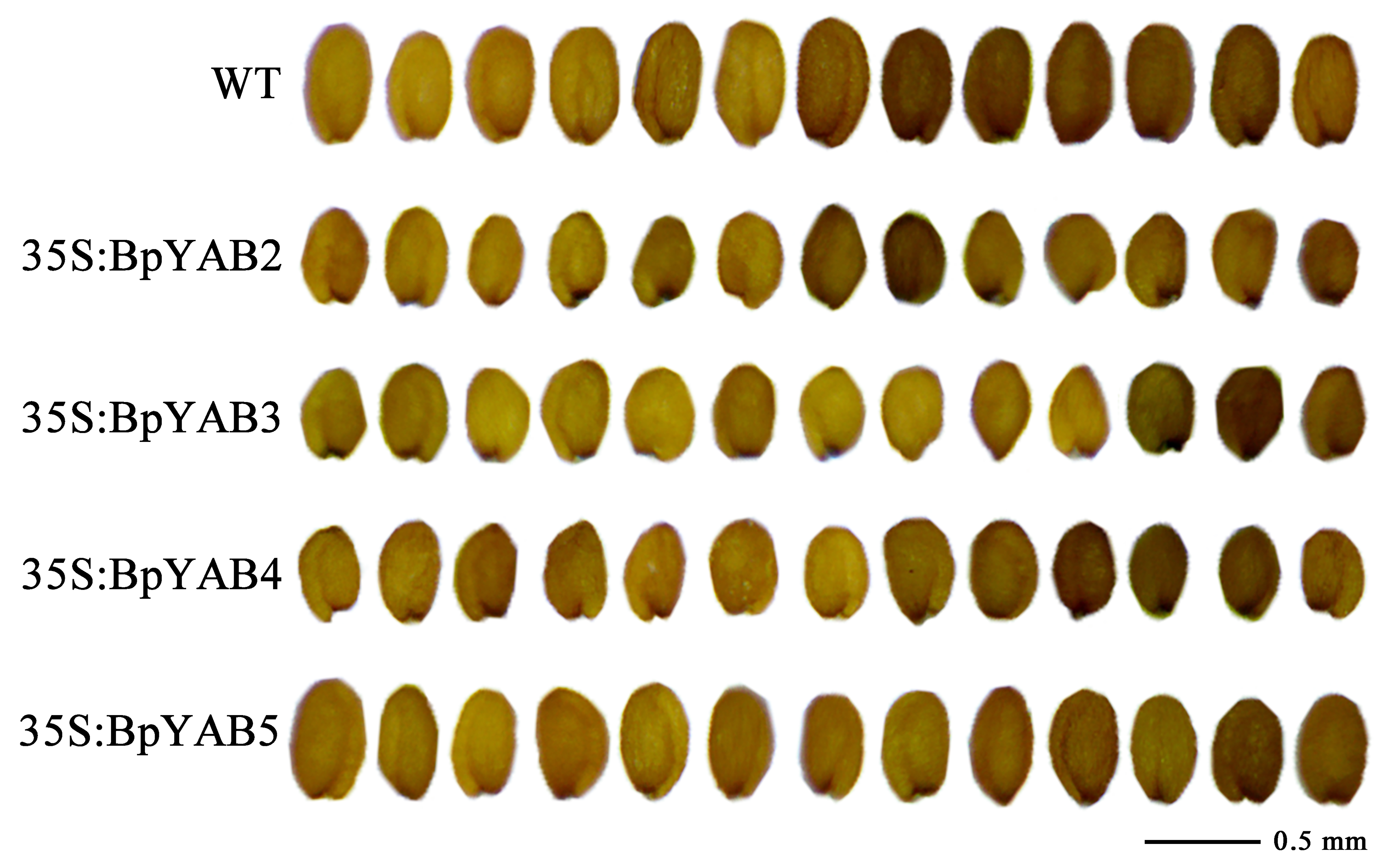

Supplement: Supplementary file 1 [file ijms-23-01670-s001.zip › Fig. S7.tif]
